# The Alteration of M6A-Tagged Transcript Profiles in the Retina of Rats After Traumatic Optic Neuropathy

**DOI:** 10.3389/fgene.2021.628841

**Published:** 2021-02-16

**Authors:** Xiaolin Qu, Kaixin Zhu, Zhenxing Li, Danfeng Zhang, Lijun Hou

**Affiliations:** ^1^ Department of Neurosurgery, Changzheng Hospital, Naval Medical University, Shanghai, China; ^2^ Department of Neurosurgery, Jinling Hospital, School of Medicine, Nanjing University, Nanjing, China

**Keywords:** traumatic optic neuropathy, N6-methyladenosine, retina, epigenetics, optic nerve

## Abstract

Messager RNA (mRNA) can be modified in a variety of ways, among which the modification of N6-methyladenosine (m6A) is one of the most common ones. Recent studies have found that the m6A modification in mRNA could functionally regulate the splicing, localization, translation, and stability of mRNA, which might be closely related to multiple diseases. However, the roles of m6A modification in traumatic optic neuropathy (TON) are unknown. Herein, we detected the expression of m6A-related genes *via* quantitative real-time PCR (qRT-PCR) and performed methylated RNA immunoprecipitation sequencing (MeRIP-seq) as well as RNA-sequencing to analyze the alteration profiles of m6A modification after TON. The results showed that the expression of m6A-related genes (METTL3, WTAP, FTO, and ALKBH5) were all upregulated after TON. In all, 2,810 m6A peaks were differentially upregulated and 689 m6A peaks were downregulated. In addition, the hypermethylated and hypomethylated profiles of mRNA transcripts were also identified. To sum up, our study revealed the differentially expressed m6A modification in the early stage of TON, which may provide novel insights into the mechanism and treatment of TON.

## Introduction

Traumatic optic neuropathy (TON) refers to a common complication of traumatic brain injury (TBI), and the incidence of TON ranges from 1.5 to 4% (Guy et al., 2014). The sufferers of TON mainly showed vision impairment and even ablepsia, bringing huge burdens to their family and the whole of society (Sosin et al., 2016; Bastakis et al., 2019). Although numerous clinical trials and fundamental research has been conducted, therapeutic strategies for TON are still limited, which may be owing to the insufficient perception of the pathophysiological mechanisms in the progression of TON (Chaon and Lee, 2015; Sosin et al., 2016). Nowadays, corticosteroids and optic nerve decompressive surgery are major treatments for TON, though neither of them could reach definitive curative effects (Emanuelli et al., 2015; Oh et al., 2018). Therefore, it is urgent to explore more effective ways to improve the vision of TON patients. More recently, noncoding RNAs (ncRNAs) and specific RNA modification, including circular RNAs (circRNAs), long-noncoding RNAs (lncRNAs), microRNAs (miRNAs), and N6-methyladenosine (m6A) modifications have been found to be expressed differentially after TBI, which provided new directions for elucidating the physiological process of TBI (Wang et al., 2019; Qu et al., 2020). However, there is no study on the relationship between m6A modification and TON. The roles of m6A-tagged transcripts in TON need to be elucidated.

Generally, there are two covalent modifications at the two termini of mRNA:7-methylguanosine cap structure and polyadenylate tail (Ramanathan et al., 2016; Stewart, 2019). Besides, a large number of nucleotide modifications have been found to be abundant in eukaryotic mRNAs, such as m6A, 5-methylcytosine (m5C), and N1-methyladenosine (m1A; Maity and Das, 2016). m6A modification is the most common one in eukaryotic mRNAs, accounting for approximately 80% of the total methylation modifications (Li et al., 2019). The development of methylated RNA immunoprecipitation sequencing (MeRIP-seq) and other technologies have increased our understanding of the role of m6A modifications in mRNAs. Regulated by methyltransferases (METTL3, METTL14, WTAP, etc.) and demethylases (FTO, ALKBH5, etc.), m6A modification is capable of participating in various fundamental biological mechanisms, for example, the regulation of gene expression, self-renewal of neural stem cells, the formation of metabolic diseases, the maintenance of biological circadian rhythms, and the development of cancers (Fischer et al., 2009; Fustin et al., 2013; Zhou et al., 2015, 2018; Maity and Das, 2016; Hsu et al., 2017).

In view of the inseparable relationship between m6A modification and protein translation, it is advisable to speculate that the dysregulation of m6A modification may be correlated with the pathophysiological processes following TON. The combination of quantitative real time-PCR (qRT-PCR), MeRIP-seq, RNA-sequencing and bioinformatic analysis were used to figure out the alteration profiles of m6A modification in the rat retina after TON. Furthermore, this study also revealed some potential roles of m6A-modified transcripts in TON.

## Materials And Methods

### Animals

The protocols for animal studies were approved by the Animal Ethics Committee of the Naval Medical University (Shanghai, China), Ethic Certificate No: 81371382. All experiments were performed under the guidance of the National Institute of Health Guide for the Care and Use of Laboratory Animals. Male Sprague-Dawley (SD) rats (220–250 g, 7–8 weeks of age) were housed for at least 7 days in a temperature-regulated (22–25°C) and humidity-controlled (50% relative humidity) animal faculty with a 12-h light/dark cycle. During the experiments, all rats had free access to water and food, except that food was withheld overnight before surgery.

### Establishment of the Experimental Traumatic Optic Neuropathy Model

In this study, the TON model was established by clamping the optic nerve of rats, as previously described (Jiang et al., 2013; Ibrahim et al., 2018; Oku et al., 2019). Briefly, adult male SD rats were intraperitoneally anesthetized with pentobarbital sodium (1%, 35 mg/kg body mass) and placed on an electric heating blanket to ensure the stabilization of body temperature at 37°C (Zhou et al., 2019). After anesthesia, microsurgical instruments were used to cut the upper eyelid skin vertically under a microscope. The optic nerve was then exposed by cutting the bulbar conjunctiva and conducting the blunt separation of retrobulbar adipose tissue. To cause the injury, the optic nerve was directly clamped at 2 mm behind the eyeball for 10 s by forceps with a 40 g constant holding force. After injury, the surgical incision was sutured, chlortetracycline eye ointment was used for anti-infection. In the sham-operated group, rats received identical surgical procedure, but without clamping applied to the optic nerve. A total of 40 rats were assigned to the TON model group and 36 to the sham group.

### The Separation and Purification of Retina

The extraction of retinal tissue was in accordance with the protocol provided by Nie et al. (2018). In brief, 12 h after operation, rats were decapitated under sterile conditions. Under the microscope, the eyeball was cut off along the limbus of the cornea, and the cornea, lens, and vitreous body were separated to obtain the retinal tissues. The separated retinal tissue was then detached by 0.125% trypsin and maintained at 37°C for 20 min. The supernatant was removed by centrifuging the digested retinal tissue at the rate of 200×*g*, followed by adding 0.25% trypsin inhibitor (T9003; Sigma-Aldrich Chemical Company, MO, United States) for neutralization and re-centrifugation. After the supernatant was discarded, the retinas were rinsed with Kreb’s solution comprising Mg^2+^ and 1% bovine serum albumin (BSA) three times. After the culture medium was added, tubularis was used to triturate and disperse the cells. The single-cell suspension was then transferred to culture dishes covered by 0.1 mg/ml of 192 poly-L-ornithine (P4538; Sigma-Aldrich Chemical Company, MO, United States) and 1 μg/ml laminin (l6274; Sigma-Aldrich Chemical Company, MO, United States). Then, Basal medium eagle (BME) was added comprised of 25 μmol/L glutamine, 10% fetal bovine serum (FBS), and 0.1 mg/ml gentamicin (SBJ-ME1268, SenBeiJia Biological Technology Co., Ltd., Jiangsu, China) and incubated at 37°C with 5% CO_2_. At last, The suspension was placed in BME medium comprised of rat Thy-1.1 antibody (ab44898; 1:1000; Abcam, Cambridge, United Kingdom) and goat anti-rat immunoglobulin G (IgG) antibody (ab150077, 1:1000; Abcam, Cambridge, United Kingdom) with incubation for 30 min at 37°C.

### Quantitative Real-Time PCR Assay

The expression levels of m6A-related genes FTO, ALKBH5, METTL3 and WTAP in the retina of injured and sham-operated rats were analyzed by using qRT-PCR. Total RNAs were extracted from the purified retina using Trizol reagent (Invitrogen, CA, United States) under the guidance of the manufacturer’s protocol. The concentration and purity of total RNA were measured by NanoDrop ND-1000 (Thermo Fisher Scientific, MA, United States). Reverse transcription was conducted using PrimeScript RT Master Mix (Perfect Real Time; Takara, Shiga, Japan) to synthesize complement DNA. The amplification of qRT-PCR was performed through pre-denaturation, 40 PCR cycles and establishing the melting curve. qRT-PCR was carried out in ProFlex 96-well PCR System (Thermo Fisher Scientific, MA, United States) with SYBR Green master mix (Applied Biosystems, CA, United States). Glyceraldehyde 3-phosphate dehydrogenase (GAPDH) was used as an internal parameter to normalize the data. Comparative threshold method (2^−*Δ*ΔCT^ method) was used to acquire relative expression levels, which were calculated according to the formula (Livak and Schmittgen, 2001):

$$\Delta \Delta \mathrm{Ct}=\Delta \mathrm{Ct}\left(\mathrm{sample}\right)-\Delta \mathrm{Ct}\left(\mathrm{calibrator}\right)$$

$$\mathrm{Relative expression}=2^{-\Delta \Delta \mathrm{CT}}$$

Where Ct, replicate-averaged threshold cycle. ∆Ct, the Ct of the target gene subtracted from the Ct of the reference gene. Each qPCR reaction was repeated three times.

The full base sequences of METTL3, ALKBH5, WTAP, FTO, and GAPDH were downloaded from NCBI in line with GenBank sequence number, and the primers were designed by Primer premier V6.0 software (Premier Biosoft International, United States). The primers used in this procedure are presented in Table 1.

**Table 1:** The sequence of primers used in real-time quantitative PCR analysis.

Name	Sequence (5'-3')	Product size (bp)	Annealing temperature (°C)
GAPDH-S	CTGGAGAAACCTGCCAAGTATG	138	60
GAPDH-A	GGTGGAAGAATGGGAGTTGCT		
FTO-S	AGAGCAGAGCAGCCTACAACGT	202	60
FTO-A	CTGGACTCGTCATCGCTTTCAT		
ALKBH5-S	TTCTTTAGCGACTCGGCACTTT	328	60
ALKBH5-A	CCTTGCGGTGGGACCTTTT		
METTL3-S	ATCCAGGCCCATAAGAAACAAC	275	60
METTL3-A	GATACAGCATCAGTGGGCAAGG		
WTAP-S	GCCTGGAAGTTTACGCCTGATA	237	60
WTAP-A	AATGGTGCTCTGCATACCCTCT		

PCR, polymerase chain reaction.

## RNA-Seq

Twelve hours after operation, rats were anesthetized with pentobarbital sodium and were transcardially perfused with 20 ml of 4°C PBS. The retinal tissues were rapidly dissected and stored in liquid nitrogen. According to the manufacturer’s protocol, total RNAs from the retina of each rat were isolated using TRIzol reagent (Invitrogen). Three replicates were conducted for the sham-operated and TON model group. To attain 60 μg of total RNA, retinal samples from four rats were integrated into one centrifuge Tube. The concentration and quality of total RNAs were assessed using NanoDrop ND-1000 (Thermo Fisher Scientific). The integrity of RNA was evaluated by denaturing agarose gel electrophoresis. The extraction of mRNA was conducted using NEBNext rRNA Depletion Kit (New England Biolabs, Hertfordshire, United Kingdom) following the manufacturer’s protocol. Bioanalyzer 2,100 system (Agilent Technologies, CA, United States) was used for quality control and quantification of the RNA libraries, and Illumina Hiseq4000 platforms (Illumina, CA, United States) was used for double-ended sequencing of RNA.

Paired-end reads were harvested from Illumina HiSeq 4,000 sequencer and were quality controlled by Q30. After 3' adaptor-trimming and low quality reads removing by cutadapt software (v1.9.3), the high quality clean reads were aligned to the reference genome (UCSC hg19) with hisat2 software (v2.0.4; Kechin et al., 2017). Then, guided by the Ensembl gtf gene annotation file, cuffdiff software (part of cufflinks) was used to get the gene level FPKM as the expression profiles of mRNA, and fold change and *p*-value were calculated based on FPKM, differentially expressed mRNA were identified (Trapnell et al., 2010).

### MeRIP-Seq, Data Analysis, and Bioinformatics

At 12 h after operation, rats were anesthetized with pentobarbital sodium and were transcardially perfused with 20 ml of 4°C PBS. The retina was then rapidly dissected and stored in liquid nitrogen. Total RNAs from the retinal tissue of each rat were isolated using TRIzol reagent (Invitrogen) according to the manufacturer’s protocol. In this study, three biological replicates were conducted in the sham-operated and TON model group, and retinal tissue samples from four rats were combined into one sample to attain 60 μg of total RNA. mRNA extraction was performed using NEBNext rRNA Depletion Kit (New England Biolabs) following the manufacturer’s protocol. Briefly, m6A RNA immunoprecipitation was performed with the GenSeq™ m6A RNA IP Kit (GenSeq Inc., shanghai, China) by following the manufacturer’s instructions. Both the input sample without immunoprecipitation and the m6A IP samples were used for RNA-seq library generation with NEBNext® Ultra II Directional RNA Library Prep Kit (New England Biolabs). The quality control (QC) of the libraries was evaluated using BioAnalyzer 2,100 system (Agilent Technologies). Library sequencing was performed on an Illumina Hiseq4000 platforms under 150 bp pair-ended resolution.

Cutadapt software (V1.9.3) was used to remove low quality reads and obtain clean reads (Kechin et al., 2017). The clean reads from all samples were then aligned to Ensembl reference genome by HISAT2 software (v2.0.4; Kim et al., 2015). Model-based Analysis of ChIP-Seq(MACS) software (v1.4.2) was used to identify methylated genes in each sample (Zhang et al., 2008). DiffReps software was used for identification of differential methylated genes (Shen et al., 2013). The differentially methylated peaks (fold changes ≥ 2.0 and *p* < 0.05) between the sham-operated group and TON model group were analyzed by exomePeak and annotated accordingly by the latest Ensembl database. Gene ontology (GO) database^1^ and the latest Kyoto Encyclopedia of Genes and Genomes (KEGG) database^2^ were used to perform GO analysis and pathway enrichment analysis on differentially methylated mRNA genes.

### Statistical Analyses

Data are expressed as mean ± standard deviation (SD). Statistical analyses were conducted using GraphPad Prism Version 8.3 software (GraphPad Software LLC, CA, United States). Student’s *t*-test (paired) was used for comparing the statistical significance between two groups. For each analysis, *p* < 0.05 was considered as statistically significant and marked with ^*^.

## Results

### m6A-Related Genes Were Upregulated in Retina After TON in Rats

**Figure 1:**
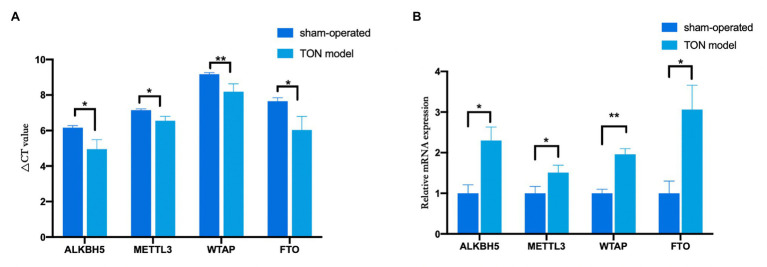
The mRNA levels of methyltransferases and demethylases in the retina tissue of rats at 12 h after TON. **(A)** Quantitative real-time PCR was used to analyze *Δ*CT value of METTL3, WTAP, ALKBH5, and FTO in the retina of rats at 12 h after TON. **(B)** mRNA levels of METTL3, WTAP, ALKBH5, and FTO at 12 h after TON. These data were normalized by level of GAPDH. Data are presented as mean ± SD. Data are expressed as mean ± SD. Data were analyzed using student’s *t*-test. ^*^p < 0.05 vs. sham group; ^**^p < 0.01 vs. sham group (*n* = 3 each). SD, standard deviation; TON, traumatic optic neuropathy.

Through qRT-PCR assay, the expression levels of four major m6A-related enzymes including METTL3, WTAP, FTO, and ALKBH5 in the retina were assessed at 12 h after the establishment of the TON model. Twelve hours after TON, mRNA levels of methyltransferases (METTL3 and WTAP) and demethylases (FTO and ALKBH5) were both significantly upregulated in the retina (Figures 1A,B; METTL3, FTO, and ALKBH5 *p* < 0.05; WTAP *p* < 0.01).

### Profiles of m6A Modification in Retina After TON

**Figure 2:**
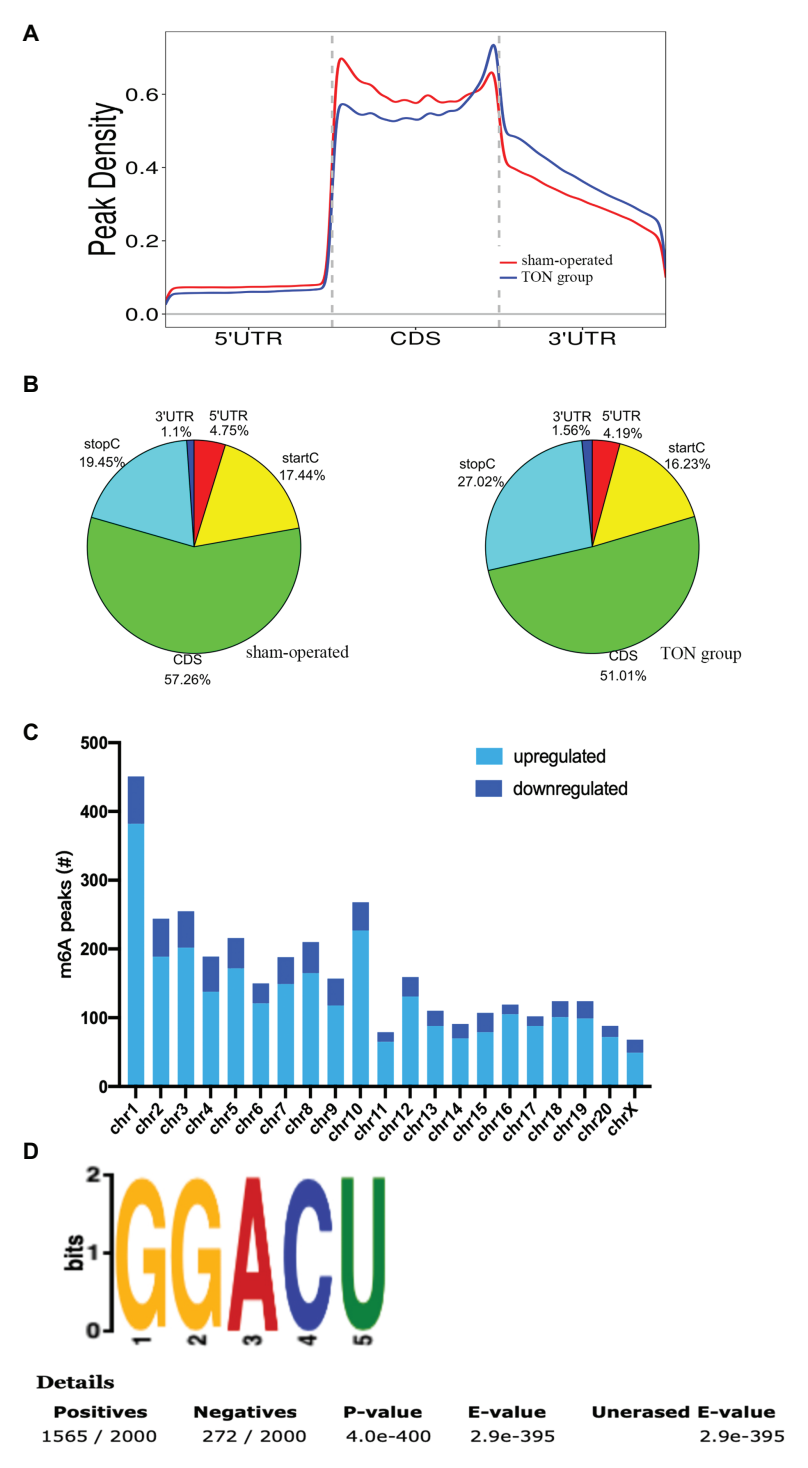
Overview of m6A modified transcripts in the retina of rats after traumatic optic neuropathy. **(A)** Metagene plots revealing the distribution of m6A peaks screened across all transcripts in the sham and TON retina. **(B)** Pie charts showing the region of m6A peaks in sham and TON groups. **(C)** The count of m6A peaks in rat chromosomes. **(D)** Top motif related to altered m6A peaks.

The alterations of m6A-tagged mRNA were assessed *via* genome-wide screening after TON in rats (Supplementary Table S1). Twelve hours after TON, a total of 2,810 m6A peaks were differentially upregulated, while 689 m6A peaks were downregulated (fold changes ≥ 2.0 and *p* < 0.05). The top 20 differentially expressed m6A peaks are listed in Table 2. These m6A peaks were enriched in coding sequence (CDS; Figures 2A,B). The differentially expressed m6A peaks can be found on all chromosomes, mainly on chr1, chr2, chr3, and chr10 (Figure 2C). Moreover, the m6A peaks were both mainly characterized by GGACU motif (Figure 2D).

**Table 2:** The top 20 transcripts with differentially expressed m6A peaks.

Gene name	Fold change	Regulation	Chromosome	Peak length	Peak start	Peak end	*p* value
Mettl24	138.7	Up	chr20	431	50,231,861	50,232,292	2.494E-09
Il1b	119.2	Up	chr3	161	128,136,841	128,137,002	1.5483E-09
Npffr1	105.7	Up	chr20	37	33,028,361	33,028,398	5.5061E-09
Afg3l2	82.6	Up	chr18	28	62,369,761	62,369,789	2.5413E-07
Cdca3	80.8	Up	chr4	183	224,369,101	224,369,284	3.3323E-07
Topaz1	77.6	Up	chr8	134	130,966,892	130,967,026	5.9663E-07
Olr1387	76.1	Up	chr10	15	34,330,521	34,330,536	5.5156E-07
Csf2rb	75.9	Up	chr7	439	119,557,641	119,558,080	4.7488E-08
Nlrp10	74.2	Up	chr1	319	180,512,001	180,512,320	1.0298E-06
Jakmip2	73.7	Up	chr18	239	37,525,101	37,525,340	1.1585E-06
Huwe1	172.5	Down	chrX	26	21,871,674	21,871,700	4.7111E-09
Lrp2	136.3	Down	chr3	131	62,312,089	62,312,220	3.8785E-09
Ints6	119.5	Down	chr15	70	49,698,721	49,698,791	1.1276E-08
Casp7	117.5	Down	chr1	85	284,614,395	284,614,480	6.6794E-11
Obsl1	100.5	Down	chr9	219	82,457,241	82,457,460	4.0605E-07
Plgrkt	85	Down	chr1	164	254,763,021	254,763,185	2.839E-06
Ints2	75.8	Down	chr10	103	73,584,338	73,584,441	5.1115E-07
Ncan	67	Down	chr16	239	20,926,001	20,926,240	9.366E-06
ST7	63.6	Down	chr4	103	45,811,420	45,811,523	5.4752E-06
Pex5	23.6	Down	chr4	109	223,443,361	223,443,470	1.0186E-06

m6A, N6-methyladenosine.

### GO Analysis and Pathway Analysis of Differentially m6A-Modified mRNA

**Figure 3:**
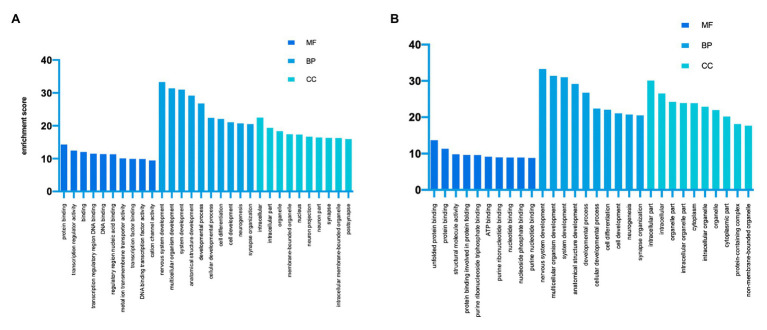
Gene Ontology enrichment of altered m6A-modified transcripts. **(A)** Top GO terms enriched across upregulated m6A-tagged transcripts. **(B)** Top GO terms enriched across downregulated m6A-tagged transcripts. MF, molecular function; BP, biological process; CC, cellular component.

GO analysis and KEGG analysis were conducted to investigate the pathophysiological significance of m6A modification. According to the GO analysis, the upregulated peaks in the TON retina were significantly associated with protein binding (ontology: molecular function), nervous system development (ontology: biological process), and intracellular (ontology: cellular component; Figure 3A). The downregulated peaks were mainly related to unfolded protein binding (ontology: molecular function), cellular localization (ontology: biological process), and intracellular part (ontology: cellular component; Figure 3B).

**Figure 4:**
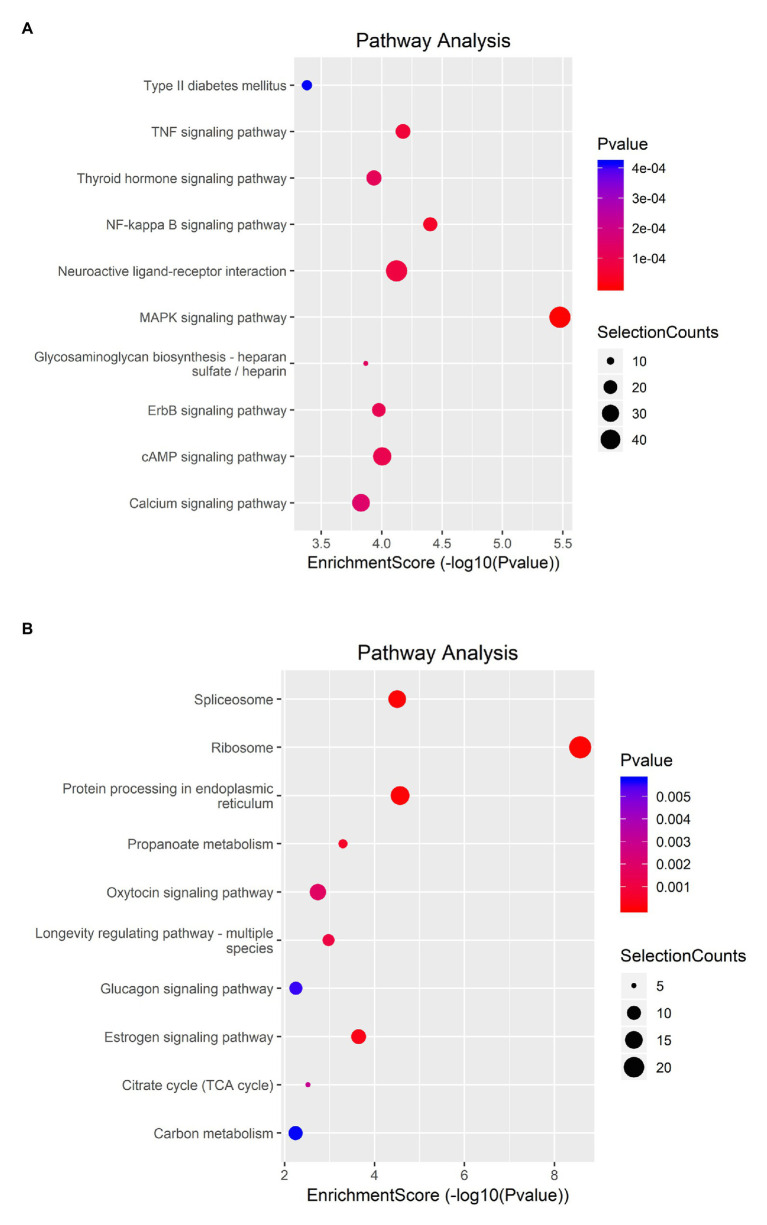
KEGG pathway analysis of m6A modified transcripts. **(A)** Top 10 KEGG terms enriched across upregulated m6A-tagged transcripts. **(B)** Top 10 KEGG terms enriched across downregulated m6A-tagged transcripts.

**Figure 5:**
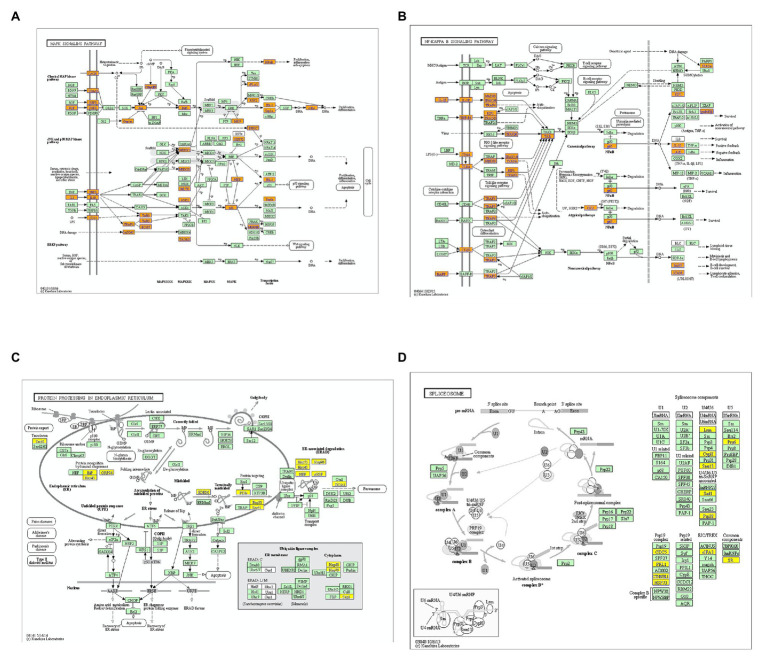
Enrichment of altered m6A-tagged transcripts in the major signaling pathways. **(A)** Enrichment of m6A-tagged transcripts in mitogen-activated protein kinase (MAPK) signaling pathways, m6A peaks in the genes highlighted with orange are up-regulated. **(B)** Enrichment of m6A-tagged transcripts in nuclear factor kappa-B (NF-*κ*B) signaling pathwaysm6A peaks in the genes highlighted with orange are up-regulated. **(C)** Enrichment of m6A-tagged transcripts in protein processing in endoplasmic reticulum pathway, m6A peaks in the genes highlighted with yellow are down-regulated. **(D)** Enrichment of m6A-tagged transcripts in spliceosome pathway, m6A peaks in the genes highlighted with yellow are down-regulated.

KEGG analysis showed that upregulated m6A peaks were remarkably associated with MAPK signaling pathway, NF-*κ*B signaling pathway, and TNF signaling pathway (Figure 4A). The downregulated m6A peaks were significantly correlated with ribosome pathway (Figure 4B). The enrichment of genes in the four major pathways is shown in Figure 5.

### Overview of mRNA Expression Profiles and Conjoint Analysis of MeRIP-Seq & RNA-Seq

**Figure 6:**
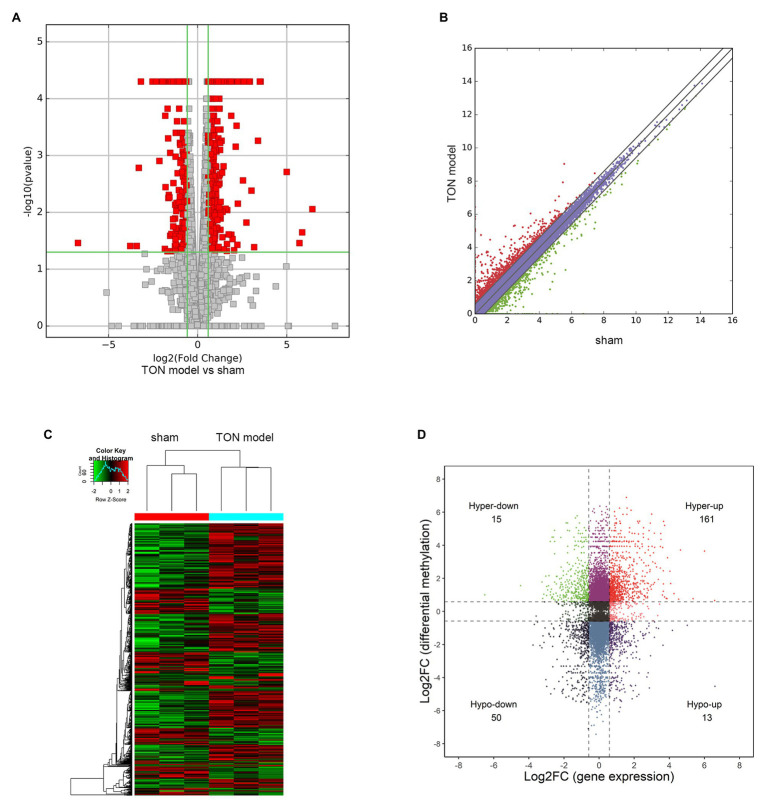
Overview and conjoint analysis of MeRIP-seq & RNA-seq of traumatic optic neuropathy retina in rats. **(A)** Volcano plots revealing the expression of mRNAs that were altered between sham and TON model with statistical significance (fold changes ≥ 2.0 and *p* < 0.05). **(B)** Scatter plot showing the differentially expressed mRNAs. **(C)** Hierarchical clustering analysis showing the differentially expressed mRNAs. **(D)** Four quadrant graph showing the differentially expressed transcripts with significant change in m6A level.

Through RNA-seq, the expression profiles of altered genes were determined (Supplementary Table S2). A total of 520 up-regulated genes and 258 down-regulated genes were detected (fold change ≥ 2.0, *p* ≤ 0.05; Figures 6A,B). The top 20 genes were shown in Table 3. The degree of differentially expressed genes between the two groups were analyzed by hierarchical cluster analysis (Figure 6C). By conducting conjoint analysis of MeRIP-seq & RNA-seq, 176 hypermethylated m6A peaks were identified in 161 upregulated mRNA transcripts (hyper-up) and 15 downregulated transcripts (hyper-down); 63 hypomethylated m6A peaks were identified in 13 upregulated mRNA transcripts (hypo-up) and 50 downregulated transcripts (hypo-down; Figure 6D).

**Table 3:** The top 20 differentially expressed mRNAs after traumatic optic neuropathy.

Gene name	Fold change	Regulation	Location	Strand	*p* value
Hnrnph3	32.49	Up	chr20:29013727-29017770	+	0.041
Rlim	23.13	Up	chrX:74567729-74583626	−	0.0001
RGD1563962	19.69	Up	chr2:185165819-185177312	+	0.0028
LOC100912538	18.76	Up	chr2:9560798-9574322	+	0.0042
Hmox1	16.42	Up	chr19:25622555-25629372	+	0.0001
Myc	14.71	Up	chr7:103157451-103162376	+	0.0007
Ccl2	14.52	Up	chr10:69047090-69048889	+	0.0152
Bcl3	13.29	Up	chr1:81996115-82010351	−	0.0362
Ckm	13.21	Up	chr1:81587879-81598112	+	0.0465
Mt2A	11.48	Up	chr19:11283093-11283867	−	0.0001
Krt5	13.57	Down	chr7:141118201-141122455	−	0.0001
Trim29	8.98	Down	chr8:46295149-46319894	+	0.0031
Krt13	8.17	Down	chr10:87824804-87828715	−	0.0001
Krt12	6.88	Down	chr10:87124673-87131666	−	0.0001
Dsp	6.22	Down	chr17:29202518-29249169	−	0.0001
Anxa8	6.03	Down	chr16:8737580-8752766	+	0.0074
Rspo1	5.92	Down	chr5:146755087-146776423	+	0.0129
Krt15	5.86	Down	chr10:87839477-87843297	−	0.0002
Sass6	4.82	Down	chr1:71220026-71232105	−	0.0262
Aqp5	4.78	Down	chrX:115753740-115757278	+	0.0005

## Discussion

m6A is the most common methylation modification in eukaryotic cells, accounting for more than 80% of methylated sites in RNA (Bodi et al., 2010). By regulating the eukaryotic transcriptome, m6A modification can affect the stability, splice, export, and translation of mRNA transcription. A variety of studies have shown that dysregulation of RNA methylation could be associated with many biological processes, including neuron development and neurodegenerative diseases (Du et al., 2019). Besides, m6A modification also plays an important role in the process of self-renewal and differentiation of neural stem cells, which may promote stem cell treatment and gene therapy for neurological diseases (Boles and Temple, 2017; Zhou et al., 2018). A recent study conducted genome-wide comparison of m6A-tagged transcript in the hippocampus of mice after TBI, and a total of 922 m6A modification sites were detected, among which 370 were up-regulated and 552 down-regulated (Wang et al., 2019). However, the role of m6A modification in TON has not been characterized. To explore the potential function of differentially expressed m6A modifications in TON, we performed MeRIP-seq to screen the profiles of m6A-tagged transcript in the retina of TON at the early stage. First, using qRT-PCR, we found that m6A-related genes (METTL3, WTAP, FTO, and ALKBH5) were both upregulated at 12 h in the retina of rats after TON. Then, MeRIP-seq was performed to determine the profiles of genome-wide m6A-tagged transcript at 12 h after TON. Briefly, 2,810 m6A peaks were differentially upregulated and 689 m6A peaks were downregulated. Furthermore, GO analysis and pathway analysis were conducted to predict the potential role of m6A modification in the pathophysiological process after TON. Finally, we applied conjoint analysis of MeRIP-seq &RNA-seq.

The “write” of m6A methylation, namely, the addition of methyl groups to the N6 position of adenosine, is catalyzed by a large methyltransferase complex whose core region contains two proteins, the heterodimer of METTL3 and METTL14. There is mounting evidence to implicate the importance of METTL3 in the nervous system. METTL3 knockout may lead to early embryonic death and impair the formation of mature neurons in embryoid bodies (Angelova et al., 2018). METTL3 mediated m6A modification may be involved in cerebellar development by regulating the stability of concerned mRNA (Wang et al., 2018). As a subunit of m6A methyltransferase, WTAP regulates the catalytic activity of methyltransferase complex by interacting with METTL3 and METTL14 (Ping et al., 2014). WTAP is over-expressed in the glioblastoma stem cells, controlling the invasion of glioblastoma cells *via* regulating epidermal growth factor (EGF) signaling (Jin et al., 2012; Xi et al., 2016). FTO and ALKBH are two major m6A RNA demethylases. FTO was firstly recognized as a member of Fe (II)‐ and oxoglutarate-dependent AlkB dioxygenase family and related to adipogenesis (Zhao et al., 2014). The deficiency of FTO could restrain the differentiation of neural stem cells *in vivo*, impairing learning and memory (Li et al., 2017). Decreasing FTO *via* microinjection of trained herpes simplex virus (HSV) vector could improve contextual fear memory (Walters et al., 2017). Li et al. (2018) have found that FTO is involved in insulin defects-related Alzheimer’s disease mice through activating TSC1-mTOR-Tau signaling. In the neurons of mice, m6A RNA demethylase ALKBH5 was recently found to be widely expressed (Du et al., 2020). During the development of the brain, ALKBH5 decreased dramatically. In brief, these studies indicated that m6A modification methyltransferases and demethylases (METTL3, WTAP, FTO, and ALKBH5) have much to do with the development of nervous system and neurological function such as learning and memory. In the present study, METTL3, WTAP, FTO, and ALKBH5 showed increased expression in the retina of mice at 12 h after TON. However, the exact roles of methyltransferases and demethylases in the pathophysiological process still need further investigation.

Nowadays, m6A modification profiles in the neurological diseases have drawn more and more attention. Liu et al. (2020) have characterized the landscape and distribution patterns of m6A and m6Am modification in the tissues of humans and mice. They identified that 594 out of 21,480 m6A peaks were tissue specific, among which the brain-specific m6A signals were closely related to the functions of head development. In transient focal ischemia model of Adult C57BL/6J mice, m6A levels were found to be increased significantly at 12 h and 24 h of reperfusion. m6A peaks were upregulated in 139 transcripts (122 mRNAs and 17 lncRNAs) and downregulated in8 transcripts (5mRNAs and 3 lncRNAs) at 12 h after reperfusion (Chokkalla et al., 2019). Yu et al. (2020) found that 2,165 m6A peaks were significantly changed in the cerebral cortex of rats after TBI, of which 1,062 were upregulated and 1,103 were downregulated. According to their results, functional FTO is of great necessity to the repair of TBI-related neurological damage. In the present study, m6A modification peaks were screened by MeRIP-seq, 2,810 m6A peaks were upregulated and 689 m6A peaks were downregulated. GO analysis showed that the altered m6A peaks mainly correlated with nervous system development, indicating that m6A RNA methylation may be crucial to the pathophysiological process after TON. On the basis of pathway analysis, m6A-tagged transcripts were mainly related to MAPK signaling pathway, NF-*κ*B signaling pathway, protein processing in endoplasmic reticulum and spliceosome. The MAPK pathway has been proven to be related to nerve injury after TBI, and the Sarm1-MAPK pathway can disturb the energy balance of axons, leading to the exhaustion of adenosine triphosphate (ATP) before complete axon injury, and promoting the progression of axon injury (Yang et al., 2015). A key pathway of inflammation in central nervous system injury is the NF-κB pathway, which regulates the expression of pro-inflammatory and pro-apoptosis genes in its active form (Howell and Bidwell, 2020). Neuroprotective effect may be achieved by regulating MAPK and NF-κB signaling pathways and thus regulating inflammatory responses (Zhang et al., 2020).

By conducting conjoint analysis of MeRIP-seq & RNA-seq, we identified the transcripts which were differentially expressed as well as hypermethylated or hypomethylated. The results of the present study revealed that m6A modifications may provide a novel insight into TON.

## Conclusion

In conclusion, our study indicated that the expression of methyltransferases and demethylases (METTL3, WTAP, FTO, and ALKBH5) are both up-regulated at 12 h after TON. Through MeRIP-seq and subsequent bioinformatics analysis, the potential functions of differentially expressed m6A modified transcripts were predicted. Furthermore, altered mRNAs with hypermethylated or hypomethylated m6A peaks were identified *via* conjoint analysis of MeRIP-seq & RNA-seq data.

## Data Availability Statement

The RNA-seq and MeRIP-seq data sets have been uploaded to the Gene Expression Omnibus repository (GEO accession number: GSE165520). All the records are now publicly-available.

## Ethics Statement

The animal study was reviewed and approved by Animal Ethics Committee of Naval Medical University (Shanghai, China).

## Author Contributions

XQ: drafting/revision of the manuscript for content, including medical writing for content, major role in the acquisition of data, study concept or design, and analysis or interpretation of data. KZ: drafting/revision of the manuscript for content, including medical writing for content, major role in the acquisition of data, and analysis or interpretation of data. ZL: drafting/revision of the manuscript for content, including medical writing for content, and analysis or interpretation of data. DZ: revision of the manuscript for content and including medical writing for content. LH: revision of the manuscript for content, including medical writing for content, and analysis or interpretation of data. All authors contributed to the article and approved the submitted version.

### Conflict of Interest

The authors declare that the research was conducted in the absence of any commercial or financial relationships that could be construed as a potential conflict of interest.

## Footnotes

^1^www.geneontology.org

^2^www.genome.jp/kegg

## References

[ref1] Angelova M. T. Dimitrova D. G. Dinges N. Lence T. Worpenberg L. Carré C.. (2018). The emerging field of Epitranscriptomics in neurodevelopmental and neuronal disorders. Front. Bioeng. Biotechnol. 6:46. 10.3389/fbioe.2018.00046, PMID: 29707539 PMC5908907

[ref2] Bastakis G. G. Ktena N. Karagogeos D. Savvaki M. (2019). Models and treatments for traumatic optic neuropathy and demyelinating optic neuritis. Dev. Neurobiol. 79, 819–836. 10.1002/dneu.22710, PMID: 31297983

[ref3] Bodi Z. Button J. D. Grierson D. Fray R. G. (2010). Yeast targets for mRNA methylation. Nucleic Acids Res. 38, 5327–5335. 10.1093/nar/gkq266, PMID: 20421205 PMC2938207

[ref4] Boles N. C. Temple S. (2017). Epimetronomics: m6A marks the tempo of corticogenesis. Neuron 96, 718–720. 10.1016/j.neuron.2017.11.002, PMID: 29144970

[ref5] Chaon B. C. Lee M. S. (2015). Is there treatment for traumatic optic neuropathy? Curr. Opin. Ophthalmol. 26, 445–449. 10.1097/ICU.0000000000000198, PMID: 26448040

[ref6] Chokkalla A. K. Mehta S. L. Kim T. Chelluboina B. Kim J. Vemuganti R. (2019). Transient focal ischemia significantly alters the mA epitranscriptomic tagging of RNAs in the brain. Stroke 50, 2912–2921. 10.1161/STROKEAHA.119.026433, PMID: 31436138 PMC6759411

[ref7] Du T. Li G. Yang J. Ma K. (2020). RNA demethylase Alkbh5 is widely expressed in neurons and decreased during brain development. Brain Res. Bull. 163, 150–159. 10.1016/j.brainresbull.2020.07.018, PMID: 32717204

[ref8] Du K. Zhang L. Lee T. Sun T. (2019). m6A RNA methylation controls neural development and is involved in human diseases. Mol. Neurobiol. 56, 1596–1606. 10.1007/s12035-018-1138-1, PMID: 29909453

[ref9] Emanuelli E. Bignami M. Digilio E. Fusetti S. Volo T. Castelnuovo P. (2015). Post-traumatic optic neuropathy: our surgical and medical protocol. Eur. Arch. Otorhinolaryngol. 272, 3301–3309. 10.1007/s00405-014-3408-5 25472815

[ref10] Fischer J. Koch L. Emmerling C. Vierkotten J. Peters T. Brüning J. C.. (2009). Inactivation of the Fto gene protects from obesity. Nature 458, 894–898. 10.1038/nature07848, PMID: 19234441

[ref11] Fustin J. -M. Doi M. Yamaguchi Y. Hida H. Nishimura S. Yoshida M.. (2013). RNA-methylation-dependent RNA processing controls the speed of the circadian clock. Cell 155, 793–806. 10.1016/j.cell.2013.10.026, PMID: 24209618

[ref12] Guy W. M. Soparkar C. N. Alford E. L. Patrinely J. R. Sami M. S. Parke R. B. (2014). Traumatic optic neuropathy and second optic nerve injuries. JAMA Ophthalmol. 132, 567–571. 10.1001/jamaophthalmol.2014.82, PMID: 24744023

[ref13] Howell J. A. Bidwell G. L. 3rd. (2020). Targeting the NF-kappaB pathway for therapy of ischemic stroke. Ther. Deliv. 11, 113–123. 10.4155/tde-2019-0075, PMID: 31928138

[ref14] Hsu P. J. Zhu Y. Ma H. Guo Y. Shi X. Liu Y.. (2017). Ythdc2 is an N-methyladenosine binding protein that regulates mammalian spermatogenesis. Cell Res. 27, 1115–1127. 10.1038/cr.2017.99, PMID: 28809393 PMC5587856

[ref15] Ibrahim A. S. Elmasry K. Wan M. Abdulmoneim S. Still A. Khan F.. (2018). A controlled impact of optic nerve as a new model of traumatic optic neuropathy in mouse. Invest. Ophthalmol. Vis. Sci. 59, 5548–5557. 10.1167/iovs.18-24773, PMID: 30480743 PMC6262644

[ref16] Jiang B. Zhang P. Zhou D. Zhang J. Xu X. Tang L. (2013). Intravitreal transplantation of human umbilical cord blood stem cells protects rats from traumatic optic neuropathy. PLoS One 8:e69938. 10.1371/journal.pone.0069938, PMID: 23940534 PMC3734232

[ref17] Jin D. -I. Lee S. W. Han M. -E. Kim H. -J. Seo S. -A. Hur G. -Y.. (2012). Expression and roles of Wilms' tumor 1-associating protein in glioblastoma. Cancer Sci. 103, 2102–2109. 10.1111/cas.12022, PMID: 22957919 PMC7659328

[ref18] Kechin A. Boyarskikh U. Kel A. Filipenko M. (2017). cutPrimers: a new tool for accurate cutting of primers from reads of targeted next generation sequencing. J. Comput. Biol. 24, 1138–1143. 10.1089/cmb.2017.0096, PMID: 28715235

[ref19] Kim D. Langmead B. Salzberg S. L. (2015). HISAT: a fast spliced aligner with low memory requirements. Nat. Methods 12, 357–360. 10.1038/nmeth.3317, PMID: 25751142 PMC4655817

[ref20] Li H. Ren Y. Mao K. Hua F. Yang Y. Wei N.. (2018). FTO is involved in Alzheimer's disease by targeting TSC1-mTOR-tau signaling. Biochem. Biophys. Res. Commun. 498, 234–239. 10.1016/j.bbrc.2018.02.201, PMID: 29501742

[ref21] Li J. Yang X. Qi Z. Sang Y. Liu Y. Xu B.. (2019). The role of mRNA mA methylation in the nervous system. Cell Biosci. 9:66. 10.1186/s13578-019-0330-y, PMID: 31452869 PMC6701067

[ref22] Li L. Zang L. Zhang F. Chen J. Shen H. Shu L.. (2017). Fat mass and obesity-associated (FTO) protein regulates adult neurogenesis. Hum. Mol. Genet. 26, 2398–2411. 10.1093/hmg/ddx128, PMID: 28398475 PMC6192412

[ref23] Liu J. e. Li K. Cai J. Zhang M. Zhang X. Xiong X.. (2020). Landscape and regulation of mA and mAm Methylome across human and mouse tissues. Mol. Cell 77, 426.e6–440.e6. 10.1016/j.molcel.2019.09.032, PMID: 31676230

[ref24] Livak K. J. Schmittgen T. D. (2001). Analysis of relative gene expression data using real-time quantitative PCR and the 2(−Delta Delta C(T)) method. Methods 25, 402–408. 10.1006/meth.2001.1262, PMID: 11846609

[ref25] Maity A. Das B. (2016). N6-methyladenosine modification in mRNA: machinery, function and implications for health and diseases. FEBS J. 283, 1607–1630. 10.1111/febs.13614, PMID: 26645578

[ref26] Nie X. G. Fan D. S. Huang Y. X. He Y. Y. Dong B. L. Gao F. (2018). Downregulation of microRNA-149 in retinal ganglion cells suppresses apoptosis through activation of the PI3K/Akt signaling pathway in mice with glaucoma. Am. J. Phys. Cell Physiol. 315, C839–c849. 10.1152/ajpcell.00324.2017, PMID: 30183321

[ref27] Oh H. -J. Yeo D. -G. Hwang S. -C. (2018). Surgical treatment for traumatic optic neuropathy. Korean J. Neurotrauma 14, 55–60. 10.13004/kjnt.2018.14.2.55, PMID: 30402419 PMC6218351

[ref28] Oku H. Kida T. Horie T. Taki K. Mimura M. Kojima S.. (2019). Tau is involved in death of retinal ganglion cells of rats from optic nerve crush. Invest. Ophthalmol. Vis. Sci. 60, 2380–2387. 10.1167/iovs.19-26683, PMID: 31141609

[ref29] Ping X. -L. Sun B. -F. Wang L. Xiao W. Yang X. Wang W. -J.. (2014). Mammalian WTAP is a regulatory subunit of the RNA N6-methyladenosine methyltransferase. Cell Res. 24, 177–189. 10.1038/cr.2014.3, PMID: 24407421 PMC3915904

[ref30] Qu X. Li Z. Chen J. Hou L. (2020). The emerging roles of circular RNAs in CNS injuries. J. Neurosci. Res. 98, 1485–1497. 10.1002/jnr.24591, PMID: 32052488

[ref31] Ramanathan A. Robb G. B. Chan S. -H. (2016). mRNA capping: biological functions and applications. Nucleic Acids Res. 44, 7511–7526. 10.1093/nar/gkw551, PMID: 27317694 PMC5027499

[ref32] Shen L. Shao N. -Y. Liu X. Maze I. Feng J. Nestler E. J. (2013). diffReps: detecting differential chromatin modification sites from ChIP-seq data with biological replicates. PLoS One 8, e65598–e65598. 10.1371/journal.pone.0065598, PMID: 23762400 PMC3677880

[ref33] Sosin M. De La Cruz C. Mundinger G. S. Saadat S. Y. Nam A. J. Manson P. N.. (2016). Treatment outcomes following traumatic optic neuropathy. Plast. Reconstr. Surg. 137, 231–238. 10.1097/PRS.0000000000001907, PMID: 26710028

[ref34] Stewart M. (2019). Polyadenylation and nuclear export of mRNAs. J. Biol. Chem. 294, 2977–2987. 10.1074/jbc.REV118.005594, PMID: 30683695 PMC6398137

[ref35] Trapnell C. Williams B. A. Pertea G. Mortazavi A. Kwan G. van Baren M. J.. (2010). Transcript assembly and quantification by RNA-Seq reveals unannotated transcripts and isoform switching during cell differentiation. Nat. Biotechnol. 28, 511–515. 10.1038/nbt.1621, PMID: 20436464 PMC3146043

[ref36] Walters B. J. Mercaldo V. Gillon C. J. Yip M. Neve R. L. Boyce F. M.. (2017). The role of the RNA demethylase FTO (fat mass and obesity-associated) and mRNA methylation in hippocampal memory formation. Neuropsychopharmacology 42, 1502–1510. 10.1038/npp.2017.31, PMID: 28205605 PMC5436121

[ref37] Wang C. -X. Cui G. -S. Liu X. Xu K. Wang M. Zhang X. -X.. (2018). METTL3-mediated m6A modification is required for cerebellar development. PLoS Biol. 16:e2004880. 10.1371/journal.pbio.2004880, PMID: 29879109 PMC6021109

[ref38] Wang Y. Mao J. Wang X. Lin Y. Hou G. Zhu J.. (2019). Genome-wide screening of altered m6A-tagged transcript profiles in the hippocampus after traumatic brain injury in mice. Epigenomics 11, 805–819. 10.2217/epi-2019-0002, PMID: 30882247

[ref39] Xi Z. Xue Y. Zheng J. Liu X. Ma J. Liu Y. (2016). WTAP expression predicts poor prognosis in malignant glioma patients. J. Mol. Neurosci. 60, 131–136. 10.1007/s12031-016-0788-6, PMID: 27370540

[ref40] Yang J. Wu Z. Renier N. Simon D. J. Uryu K. Park D. S.. (2015). Pathological axonal death through a MAPK cascade that triggers a local energy deficit. Cell 160, 161–176. 10.1016/j.cell.2014.11.053, PMID: 25594179 PMC4306654

[ref41] Yu J. Zhang Y. Ma H. Zeng R. Liu R. Wang P.. (2020). Epitranscriptomic profiling of N6-methyladenosine-related RNA methylation in rat cerebral cortex following traumatic brain injury. Mol. Brain 13:11. 10.1186/s13041-020-0554-0, PMID: 31992337 PMC6986156

[ref42] Zhang C. Chen S. Zhang Z. Xu H. Zhang W. Xu D.. (2020). Asiaticoside alleviates cerebral ischemia-reperfusion injury via NOD2/mitogen-activated protein kinase (MAPK)/nuclear factor kappa B (NF-kappaB) signaling pathway. Med. Sci. Monit. 26:e920325. 10.12659/MSM.920325, PMID: 32006420 PMC7009775

[ref43] Zhang Y. Liu T. Meyer C. A. Eeckhoute J. Johnson D. S. Bernstein B. E.. (2008). Model-based analysis of ChIP-Seq (MACS). Genome Biol. 9, R137–R137. 10.1186/gb-2008-9-9-r137, PMID: 18798982 PMC2592715

[ref44] Zhao X. Yang Y. Sun B. -F. Shi Y. Yang X. Xiao W.. (2014). FTO-dependent demethylation of N6-methyladenosine regulates mRNA splicing and is required for adipogenesis. Cell Res. 24, 1403–1419. 10.1038/cr.2014.151, PMID: 25412662 PMC4260349

[ref45] Zhou Z. B. Du D. Chen K. Z. Deng L. F. Niu Y. L. Zhu L. (2019). Differential expression profiles and functional predication of circular ribonucleic acid in traumatic spinal cord injury of rats. J. Neurotrauma 36, 2287–2297. 10.1089/neu.2018.6366, PMID: 30681027

[ref46] Zhou J. Wan J. Gao X. Zhang X. Jaffrey S. R. Qian S. -B. (2015). Dynamic m(6)A mRNA methylation directs translational control of heat shock response. Nature 526, 591–594. 10.1038/nature15377, PMID: 26458103 PMC4851248

[ref47] Zhou H. Wang B. Sun H. Xu X. Wang Y. (2018). Epigenetic regulations in neural stem cells and neurological diseases. Stem Cells Int. 2018:6087143. 10.1155/2018/6087143, PMID: 29743892 PMC5878882

